# Circulating TRAIL Shows a Significant Post-Partum Decline Associated to Stressful Conditions

**DOI:** 10.1371/journal.pone.0027011

**Published:** 2011-12-14

**Authors:** Giorgio Zauli, Lorenzo Monasta, Erika Rimondi, Liza Vecchi Brumatti, Oriano Radillo, Luca Ronfani, Marcella Montico, Giuseppina D'Ottavio, Salvatore Alberico, Paola Secchiero

**Affiliations:** 1 Institute for Maternal and Child Health, IRCCS “Burlo Garofolo”, Trieste, Italy; 2 Department of Morphology and Embryology and LTTA Centre, University of Ferrara, Ferrara, Italy; State Key Laboratory of Reproductive Biology, Institute of Zoology, Chinese Academy of Sciences, China

## Abstract

**Background:**

Since circulating levels of TNF-related apoptosis inducing ligand (TRAIL) may be important in the physiopathology of pregnancy, we tested the hypothesis that TRAIL levels change at delivery in response to stressful conditions.

**Methods/Principal Findings:**

We conducted a longitudinal study in a cohort of 73 women examined at week 12, week 16, delivery and in the corresponding cord blood (CB). Serum TRAIL was assessed in relationship with maternal characteristics and to biochemical parameters. TRAIL did not vary between 12 (67.6±27.6 pg/ml, means±SD) and 16 (64.0±16.2 pg/ml) weeks' gestation, while displaying a significant decline after partum (49.3±26.4 pg/ml). Using a cut-off decline >20 pg/ml between week 12 and delivery, the subset of women with the higher decline of circulating TRAIL (41.7%) showed the following characteristics: i) nullipara, ii) higher age, iii) operational vaginal delivery or urgent CS, iv) did not receive analgesia during labor, v) induced labor. CB TRAIL was significantly higher (131.6±52 pg/ml) with respect to the corresponding maternal TRAIL, and the variables significantly associated with the first quartile of CB TRAIL (<90 pg/ml) were higher pre-pregnancy BMI, induction of labor and fetal distress. With respect to the biochemical parameters, maternal TRAIL at delivery showed an inverse correlation with C-reactive protein (CRP), total cortisol, glycemia and insulin at bivariate analysis, but only with CRP at multivariate analysis.

**Conclusions:**

Stressful partum conditions and elevated CRP levels are associated with a decrease of circulating TRAIL.

## Introduction

TNF-related apoptosis inducing ligand (TRAIL) is a TNF family member expressed as either a type II transmembrane protein or, similarly to other membrane-bound ligands of the TNF superfamily, as a soluble protein, which is detectable in the serum under physiological conditions [1]. Although the best characterized biological activity of TRAIL, also known as Apo2 ligand, is represented by a potent induction of apoptosis in a variety of cancer cell types [2], the wide expression of TRAIL and TRAIL receptors in many normal tissues suggests that the physiological role of TRAIL is more complex than merely activating the apoptotic pathway in cancer cells. Indeed, accumulating evidence suggests possible non-apoptotic functions regulated by TRAIL [3] and, in particular, by soluble TRAIL, which is able to activate intracellular signal transduction pathways involved in cell survival, migration and proliferation in a variety of normal cells [3]. These biological activities of soluble TRAIL are observed at concentrations (10–100 pg/ml) in the range found in serum/plasma [4]–[10]. Among different tissues, it is noteworthy that TRAIL and its receptors are abundantly expressed in the human placenta [11]–[14], where TRAIL has been proposed to contribute to the establishment of immune privilege during pregnancy [11], [12]. In addition, soluble TRAIL displays regulatory effects on endothelial cells and more in general on the vascular system [15]–[18]. While there are data regarding the serum level of the TRAIL receptor osteoprotegerin (OPG) [19] during pregnancy [20], anything is known about potential modulation of circulating soluble TRAIL concentrations from early pregnancy to post-partum after labor.

On these bases, the aim of the present study was to measure the serum levels of TRAIL in a cohort of women, at early time points of pregnancy (12 and 16 weeks), at the delivery, as well in the respective umbilical cord blood (CB), in order to assess: i) the relationship between TRAIL and clinical and biochemical parameters, ii) the relationship between the maternal and CB levels of circulating TRAIL.

## Materials and Methods

### Study population

The study population consisted of 73 women who underwent amniocentesis. The procedures followed were in accordance with the Declaration of Helsinki and approved by the institutional review board (Institute for Maternal and Child Health, IRCCS Burlo Garofolo of Trieste). All participant subjects gave written informed consent. Serial blood samples were obtained from each woman at 12 (time 1) and 16 (time 2) weeks' gestation and within 15 minutes post-partum (time 3). In addition, blood samples were collected as umbilical mixed arterial-venous CB samples at delivery. The samples were immediately centrifuged, aliquoted and the sera were frozen at −80°C until biochemical measurements.

The main characteristics of the 73 women are reported in **Table 1** and include: i) maternal data, such as maternal age at delivery, pre-gestational BMI (body mass index), ethnicity, parity, pregnancy, type of conception, smoking at 12 weeks' gestation, pathological course of pregnancy, gestational hypertensive disorders, gestational diabetes, karyotyping, fetal growth restriction during pregnancy; ii) delivery data, such as gestational age at delivery, induction of labor, delivery modality, such as spontaneous vaginal, operative vaginal, elective cesarean section (CS), urgent CS, analgesia during labor; iii) neonatal outcome: live births, cord blood pH and base excess (BE), Apgar at 1^st^ and 5^th^ minute, need of resuscitation in first 30 minutes after delivery, need of admission to intensive neonatal care, fetal distress. Demographic and clinical characteristics were ascertained from baseline interview, medical records/database and/or clinical medical examination. For some of the parameters reported in **Table 1** and analyzed in **Table S1**, we had some missing data: when the number of observation is less than 73 women, the number of observation is specified in **Tables 1**.

**Table 1:** Main characteristics of the enrolled women*.

Characteristics	Number (%) or Mean (±SD)
Maternal age at delivery (years)	37.9 (2.8±SD)
Parity: Nulliparous/Pluriparous	35 (47.9%)/38 (52.1%)
Pre pregnancy BMI	22.9 (4.0±SD)
BMI categories: BMI<30/BMI≥30	69 (94.5%)/4 (5.5%)
Conception: Spontaneous/Artificial	71 (97.3%)/2 (2.7%)
Ethnicity: Caucasian/Other	71 (97.3%)/2 (2.7%)
Smoking at 12 weeks gestation: No/Yes	71 (97.3%)/2 (2.7%)
Pathological course of pregnancy: No/Yes	61 (83.6%)/12 (16.4%)
Gestational Hypertensive disorders: No/Yes	68 (93.2%)/5 (6.8%)
Diabetes: No	70 (95.9%)
Pregestational diabetes/Gestational diabetes	0 (0%)/3 (4.1%)
Karyotyping (n = 67): Normal/Abnormal	67 (100%)/0 (0%)
Fetal growth restriction above 10%: No/Yes	41 (56.2%)/32 (43.8%)
Gestational age at delivery (weeks)	39 (1.5±SD)
Induction of labor (n = 72): No/Yes	59 (81.9%)/13 (18.1%)
Delivery modality:	
- Spontaneous vaginal	54 (74.0%)
- Elective CS	6 (8.2%)
- Urgent CS	8 (11.0%)
- Operative vaginal	5 (6.8%)
Analgesia during labor: Yes/No	21 (30%)/50 (70%)
Delivery outcome: Live births	73 (100.0%)
Umbilical pH (n = 62): pH≥7.1/pH<7.1	60 (96.8%)/2 (3.2%)
Umbilical BE (n = 60): BE≥−12/BE<−12	59 (98.3%)/1 (1.7%)
Apgar 1^st^ minute: Apgar≥5/Apgar<5	71 (97.3%)/2 (2.7%)
Apgar 5^th^ minute: Apgar≥7/Apgar<7	72 (98.6%)/1 (1.4%)
Need of resuscitation in first 30 min. (n = 71): No/Yes	69 (97.2%)/2 (2.8%)
Admission to Intensive Neonatal Care: No/Yes	65 (89%)/8 (11%)
Fetal distress: No/Yes	64 (88%)/9 (12%)

*n = 73 unless specified. SD: standard deviance; BMI: Body Mass Index; CS: caesarean section; BE: base excess.

### Biochemical measurements

For the measurement of circulating TRAIL, analyses were performed in duplicate by using specific, commercially available ELISA kit (R&D Systems, Minneapolis, MN) in accordance with the manufacturer's instructions and analyzed with an ELISA reader at 450 nm, as previously described [21]. Standard curves were set up by using scalar dilution series of the recombinant human proteins (TRAIL) provided by the manufacturers as standard for each specific kit. Sensitivity of the assay was 2.86 pg/ml and the intra- and inter-assay coefficients of variation (CV) were 3.9% and 6% respectively, and the upper limit of detection was 1000 pg/ml. Selected serum samples were run in each ELISA plate, as internal controls, confirming the reproducibility of the determinations over times.

Total cortisol and insulin levels were measured by Cobas Modular E analyzer (Roche Diagnostics, Basel, Switzerland) with electrochemiluminescence assay following manufacturer's instructions. For total cortisol, sensitivity of the assay was 0.018 µg/dl and the intra- and inter-assay CV were 1.3% and 2.89% respectively, and the upper limit of detection was 63.4 µg/dl. For insulin, sensitivity of the assay was 0.200 µU/ml and the intra- and inter-assay CV were 1.9% and 2.7% respectively, and the upper limit of detection was 1000 µU/ml. CRP levels were determined on Cobas Modular P analyzer (CRPL3 Tina-quant C-Reactive Protein Gen. 3 by Roche Diagnostics) immunoturbidimetric method following manufacturer's instructions. For CRP sensitivity of the assay was 0.03 mg/dl and the intra- and inter-assay CV were 1.3% and 2.43% respectively, and the upper limit of detection was 35 mg/dl. Serum glucose was measured by Cobas Modular A analyzer (Roche Diagnostics) with enzymatic methods following manufacturer's instructions. Sensitivity of the assay was 2 mg/dl and the intra- and inter-assay CV were 1.0% and 1.7%, respectively and the upper limit of detection was 750 mg/dl. All biochemical markers were determined in duplicate and were run in the same assay in each period.

### Statistical analyses

The differences among the TRAIL, total cortisol, glucose, insulin, CRP values across study phases were measured with the non-parametric Friedman test for matched samples. A post-hoc test, the non-parametric Wilcoxon signed-rank test, was then applied with Bonferroni adjustment for the number of one to one comparisons. Correlations between TRAIL and variables were estimated using Spearman's correlation coefficient.

A bivariate analysis was conducted to study the relation between difference in TRAIL values between time 1 and time 3 (higher than 20 pg) and variables related to maternal characteristics, delivery and neonatal outcomes, illustrated in **Table 1**. P values were calculated using the Mann-Whitney non parametric test in case of continuous variables and the two tailed Fisher exact test in case of dichotomous variables. Multivariate logistic regression analyses were run with TRAIL (either from maternal measurements or CB values, as detailed in the Results section) and including as potential predicting variables those related to maternal characteristics, pregnancy, delivery and neonatal outcomes. The logistic regression also considered the levels of CPR (≤0.5 vs. >0.5 mg/dl for maternal blood and ≤0.35 vs. >0.35 for CB) and total cortisol, insulin, and glucose (expressed as quartiles) at delivery and into the CB. Stata/IC 11.2 was used for the analyses (StataCorp LP, College Station, USA; www.stat.com). A two-sided p-value <0.05 has been chosen as statistically significant.

## Results

### The circulating levels of maternal TRAIL are significantly decreased immediately after partum

In **Table 1** we report the results of the descriptive analysis. Maternal age appears to be quite high due to the nature of our study, which focused on women who would accept to carry out amniocentesis. In our group of 73 women, 48% were nulliparous, four were obese (BMI≥30), two conceived with artificial methods, two were not Caucasian, two smoked during pregnancy (smoked at 12 weeks gestation). All women had a normal karyotype, while 12 had a pathological course of pregnancy.

**Figure 1:**
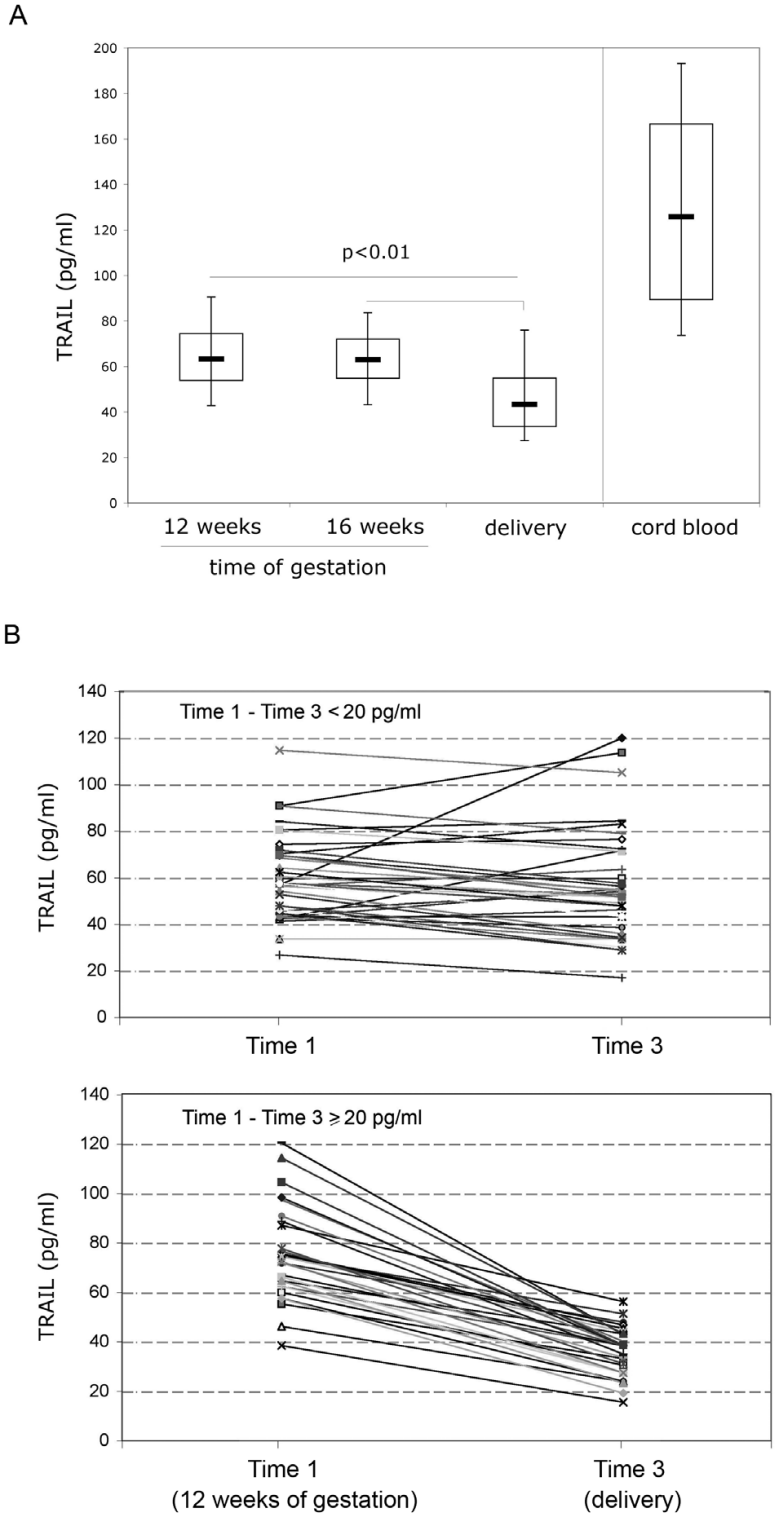
Serum TRAIL levels during pregnancy and in the corresponding cord blood. In **A**, levels of TRAIL were determined by ELISA in sera from 73 pregnant women (analyzed at the indicated times of gestation and at delivery) and from the corresponding cord blood. Horizontal bars are median, upper and lower edges of box are 75th and 25th percentiles, lines extending from box are 10th and 90th percentiles. In **B**, two groups of women enrolled in this study were identified on the basis of the drop in the TRAIL serum levels between 12 weeks of gestation (time 1) and delivery (time 3) <20 pg/ml (group 1) or ≥20 pg/ml (group 2). Individual values for each of the 73 women are shown at time 1 and time 3.

For each subject, the serum levels of TRAIL were measured in 2 different time points of the gestational period (time 1: 12 weeks, time 2: 16 weeks), at the partum (time 3) as well as in the serum derived from the corresponding CB. As shown in **Figure 1A**, the values of circulating soluble TRAIL were not significantly different between time 1 and time 2 (p = 0.703), while they showed a clear decrease at delivery (p<0.01). The non-parametric Friedman test for matched samples on the three values has a p<0.001. The Wilcoxon signed-rank test with Bonferroni correction for multiple comparisons (three comparisons: significant if p<0.017) showed significant differences between TRAIL measures at time 1 and time 3 (p<0.001), and between measures at time 2 and time 3 (p<0.001). On the other hand, the circulating TRAIL levels in the umbilical CB were significantly higher than the maternal levels examined in the different time points (in all cases p<0.0001).

By analyzing the kinetics of TRAIL at different time points, two groups of women could be identified: one group included women characterized by either no significant decrease or (in 7 cases) by an increase of TRAIL (**Figure 1B**, upper panel), while in the second group a clear-cut decline was observed (**Figure 1B**, lower panel). In particular, a subset of women (41.7%) had a decrease in TRAIL from time 1 to time 3 >20 pg/ml, showing a decrease in the levels of circulating TRAIL similar to that previously observed in patients affected by coronary artery disease [5]–[9].

### The decline in the maternal levels of circulating TRAIL in a subset of women is associated with difficult labor

In the bivariate analysis for the study of the factors associated with the outcome “TRAIL at time 1 - TRAIL at time 3” >20 pg/ml, it was observed that the women more at risk of a significant decrease in TRAIL at the partum were those: with higher age, nulliparous, with induction of labor, with operative vaginal delivery or urgent CS, who did not have analgesia during labor or with fetal distress (**Table S1**). A multivariate logistic regression analysis was run with the above mentioned outcome and including as potential predicting variables only those related to maternal characteristics, pregnancy and delivery, as reported in **Table 1**. We excluded type of conception from the multivariate logistic regression model because no women had an artificial conception in the group with reduction of TRAIL values ≤20 pg/ml. In multivariate logistic regression analysis (**Table 2**), the variables significantly (p<0.05) associated with the outcome are the following: higher maternal age at delivery, no analgesia during labor, nulliparity, induction of labor, operative vaginal delivery or urgent CS.

**Table 2:** Multivariate logistic regression analysis of factors associated with the outcome: TRAIL (T.1–T.3) >20 pg.

	OR	95% CI	p*
Maternal age at delivery	1.564	1.163–2.104	**0.003**
Pre pregnancy BMI	0.952	0.786–1.152	0.612
Nulliparity			
- No	ref	-	
- Yes	7.576	1.393–41.667	**0.019**
Ethnicity			
- Caucasian	ref	-	
- Other	138.004	0.264–72103.070	0.123
Smoke			
- No	ref	-	
- Yes	0.087	0.000–18.798	0.373
Gestational hypertensive disorders			
- No	ref	-	
- Yes	0.011	0.000–0.823	0.051
Gestational diabetes			
- No	ref	-	
- Yes	0.043	0.000–3.139	0.151
Pathological course of pregnancy			
- No	ref	-	
- Yes	34.079	0.844–1376.167	0.061
Induction of labor			
- No	ref	-	
- Yes	15.737	1.280–193.405	**0.031**
Delivery modality			
- Spontaneous vaginal or elective CS	ref		
- Urgent CS or operative vaginal	6.783	1.021–45.069	**0.048**
Fetal growth restriction above 10%			
- No	ref	-	
- Yes	4.868	0.862–27.482	0.073
Analgesia during labor			
- Yes	ref		
- No	9.346	1.709–50.549	**0.010**
Gestational age at delivery	1.818	0.955–3.464	0.069

n = 70, Pseudo R2 = 0.425. OR: Odds Ratio; CI: Confidence Interval; *p value<0.05 in bold; BMI: Body Mass Index; CS: caesarean section.

### Maternal conditions and fetal distress also affect CB TRAIL levels

In the next group of experiments, we sought to investigate whether the maternal characteristics, which are associated with the decline of maternal TRAIL at delivery, also affected CB TRAIL. For this purpose, in the absence of references for normal/pathological levels of funicular TRAIL, we decided to adopt the cut-off identifying 25% of sample with the lowest levels of funicular TRAIL. Given the size of the sample, 25% guaranteed a good size in the affected group to allow detection of possible associations with other factors. In a multivariate logistic regression model with the first quartile of CB TRAIL as outcome (0 if ≥90 pg/ml, 1 if <90 pg/ml), we could reconsider the variable on type of conception and had to exclude, however, ethnicity, smoke and diabetes mellitus because of collinearity. The variables significantly associated with a CB TRAIL <90 pg/ml were the following: higher pre-pregnancy BMI, induction of labor and fetal distress (**Table 3**).

**Table 3:** Multivariate logistic regression analysis of factors associated with the outcome: cord blood TRAIL-first quartile.

	OR	95% CI	p*
Maternal age at delivery	0.982	0.740–1.303	0.899
Pre pregnancy BMI	0.742	0.560–0.985	**0.039**
Parity			
- Nulliparous	Ref	-	
- Pluriparous	2.52	0.478–13.228	0.276
Gestational hypertensive disorders			
- No	Ref	-	
- Yes	22.485	0.0.527–959.462	0.104
Pathological course of pregnancy			
- No	Ref	-	
- Yes	0.113	0.005–2.452	0.165
Induction of labor			
- No	Ref	-	
- Yes	15.505	1.931–124.457	**0.010**
Delivery modality			
- Spontaneous vaginal or elective CS	Ref	-	
- Urgent CS or operative vaginal	0.646	0.039–10.745	0.761
Foetal growth restriction above 10%			
- No	Ref	-	
- Yes	0.956	0.241–3.789	0.949
Analgesia during labor			
- Yes	Ref	-	
- No	0.954	0.198–4.587	0.953
Gestational age at delivery	0.905	0.518–1.582	0.726
Admission to Intensive Neonatal Care			
- No	Ref	-	
- Yes	0.522	0.039–7.050	0.625
Foetal distress			
- No	Ref	-	
- Yes	37.091	0.979–1405.412	**0.049**

n = 69, Pseudo R2 = 0.234. OR: Odds Ratio; CI: Confidence Interval; *p value<0.05 in bold; BMI: Body Mass Index; CS: caesarean section.

### Maternal TRAIL levels at delivery are inversely related to CRP

**Figure 2:**
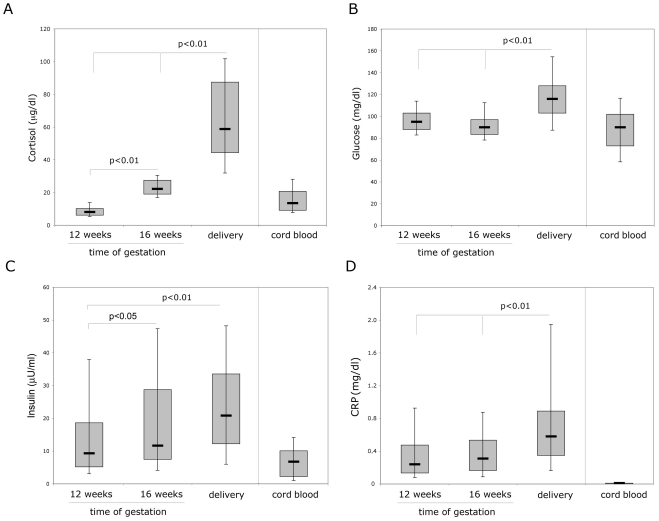
Changes in the serum levels of biochemical markers during pregnancy and in the corresponding cord blood. Serum levels of (**A**) total cortisol, (**B**) glycemia, (**C**) insulin, (**D**) CRP were determined by ELISA in sera from 73 pregnant women (analyzed at the indicated times of gestation and at delivery) and from the corresponding cord blood. Horizontal bars are median, upper and lower edges of box are 75th and 25th percentiles, lines extending from box are 10th and 90th percentiles.

Since the data illustrated above suggested that stressful conditions might be involved in mediating the decline of TRAIL observed at delivery and might also influence CB TRAIL levels, we have then investigated the potential correlation between levels of circulating TRAIL and relevant biochemical parameters, such as total cortisol, glycemia, insulinemia and CRP. In particular, CRP was included since previous studies have shown that it increases during pregnancy under both normal and pathological conditions [22]–[25]. As reported in **Figure 2** and **Table S2**, the levels of total cortisol, glycemia, insulin and CRP showed a significant increase at the delivery (time 3) with respect to the previous time points analyzed (time 1 and/or time 2). In particular, while insulin and total cortisol showed a progressive increase from time 1 to time 3 (**Figure 2** and **Table S2**), glycemia and CRP showed a peak at delivery (**Figure 2** and **Table S2**). Moreover, while the levels of total cortisol glycemia and insulin in CB were not significantly different from maternal values at times 1 and 2, the levels of CRP in CB were either completely absent or very low as compared to maternal CRP values determined at different time points (**Figure 2** and **Table S2**). It is also noteworthy that while the levels of maternal cortisol, glycemia and insulin showed a significant positive correlation with CB cortisol, glycemia and insulin (**Table 4**), no significant correlation was observed between maternal (time 3) and CB levels of TRAIL (R = 0.032, p = 0.792) nor between maternal and CB CRP (**Table 4**). Of note, a significant inverse correlation was observed between maternal TRAIL at delivery (time 3) and total cortisol, TRAIL (time 3) and glycemia, TRAIL (time 3) and insulin as well as between TRAIL (time 3) and CRP (**Table 4** and **Figure S1**). These findings were confirmed in a bivariate analysis with TRAIL time 3-first quartile as outcome (data not shown). However, in a multivariate logistic regression analysis with the outcome: TRAIL time 3-first quartile, the only biochemical variable significantly (p = 0.003) associated with a TRAIL time 3-first quartile was CRP (**Table S3**). On the other hand, glycemia, insulin and total cortisol lost of significance with the only addition of another variable (data not shown). The prominent association of CRP with maternal TRAIL was further underlined by comparing the group of women with reduction of TRAIL values ≤20 pg/ml vs the group with reduction of TRAIL values >20 pg/ml, illustrated in **Figure 1B**, for the levels of CRP at delivery. In fact, significantly (p<0.01) higher levels of CRP (median 0.79, mean±SD 1.39±1.60 mg/dl, time 3) were measured in the group of women with reduction of TRAIL values >20 pg/ml with respect to the other group (median 0.47, mean±SD 0.57±0.45 mg/dl, time 3).

**Table 4:** Correlation among the circulating levels of TRAIL and metabolic markers, as analyzed by Spearman Rank correlation Test.

Variables	TRAIL*Cord blood*	Cortisol(T.3)	Glucose(T.3)	Insulin(T.3)	CRP(T.3)
**TRAIL** **(T.3)**	R = 0.032p = 0.792	R = −0.388p = 0.001*	R = −0.281p = 0.018*	R = −0.239p = 0.045*	R = −345 p = 0.003*
**Cortisol** ***Cord blood***	R = −0.118p = 0.323	R = 0.601p = 0.000*	R = 0.485 p = 0.000*	R = 0.476 p = 0.000*	R = 0.230 p = 0.054
**Glucose** ***Cord blood***	R = −0.029p = 0.809	R = 0.549p = 0.000*	R = 0.765p = 0.000*	R = 0.519 p = 0.000*	R = 0.121 p = 0.312
**Insulin** ***Cord blood***	R = −0.119p = 0.325	R = 0.182p = 0.132	R = 0.294 p = 0.014*	R = 0.316 p = 0.008*	R = 0.586 p = 0.000*
**CRP** ***Cord blood***	R = −0.176p = 0.138	R = −0.127 p = 0.290	R = −0.070 p = 0.558	R = −0.123 p = 0.302	R = −0.109 p = 0.363

R, coefficient of correlation;

*, p<0.05.

Finally, when considering CB TRAIL, no correlations were observed between CB TRAIL and CB levels of total cortisol, glycemia, insulin and CRP (data not shown). Interestingly, when CB serum samples were subdivided on the basis of the pathological levels of glycemia (>96 mg/dl), insulin (>20 µU/ml), CRP (>0.035 mg/dl), total cortisol (>17 mg/ml), CB TRAIL levels tended to be lower in patients with high levels of total cortisol compared to those with normal levels of cortisol (p = 0.08), while no significant differences were noticed in CB samples with different levels of glycemia, insulin or CRP (**Figure S2**).

## Discussion

In this study, we have demonstrated for the first time that the circulating levels of soluble TRAIL are significantly decreased at delivery as compared to week 12 and week 16 of gestation in a group of 73 women. A subset of these women (41.7%) had a decrease in TRAIL at delivery >20 pg/ml with respect to the levels observed at week 12. Interestingly, a comparable drop in the levels of circulating TRAIL was previously observed in patients affected by acute coronary artery disease [5]–[9], a finding that led us and other Authors to hypothesize that TRAIL might have a vasoprotective activity. In keeping with the idea that physiological levels of circulating TRAIL has trophic effects on the blood vessels [15]–[18], we have demonstrated for the first time that the soluble TRAIL levels in CB were much greater than those found in their mothers as well as in the general adult population [21]. In addition, while the levels of total cortisol and glycemia and to a lesser extent insulin showed a significant positive correlation between maternal blood at delivery (time 3) and CB, no such correlation was observed for TRAIL. This lack of correlation between maternal and CB, together with the fact that TRAIL levels were much higher in CB than in maternal blood strongly suggests that TRAIL is autonomously produced also by fetal tissues or annexes. In this respect, although no information is currently available concerning the cellular source(s) of circulating TRAIL in adults, it is noteworthy that several studies have documented the presence of TRAIL, as well as of its receptors, in human placenta [11]–[14], [26]–[31]. The syncytiotrophoblast, cytotrophoblast, stromal cells and the capillary endothelium cells in human placenta all appeared to be TRAIL immunoreactive [11]–[14], [26]–[31]. However, the potential contribution of placenta for explaining both the differences between maternal and CB levels of TRAIL, as well as the drop of circulating TRAIL after delivery, remain to be established. In addition, further studies are required in order to evaluate whether TRAIL is suitable to be a marker of the health of the placenta and/or uteri during pregnancy.

Another important conclusion of our study was that a woman is more at risk of a decrease >20 pg in TRAIL between 12 weeks gestation and delivery if she is older, if she has an operational vaginal delivery or an urgent CS, if she does not receive analgesia during labor and if her labor is induced, as evaluated by dichotomous and multivariate regression analyses. In addition, by generating a dichotomous variable below or above 90 pg/ml in the CB (first quartile), it was possible to demonstrate that levels of TRAIL below 90 pg/ml in the CB were associated to higher pre-pregnancy BMI, induction of labor and fetal distress. Thus, all stressful conditions linked to partum modalities induced a significant drop in the levels of circulating TRAIL. This notion was strengthened by the observation that circulating TRAIL at delivery was inversely related with biochemical markers of stress, such as elevated glycemia, insulin and total cortisol levels, which reach the highest levels at delivery [32]. Interestingly, elevated levels of total cortisol were associated with low levels of TRAIL also in the CB although this association did not reach statistical significance. In addition, a previous study demonstrated that pathological levels of cortisol increase the circulating levels of osteoprogerin, a neutralizing receptor for TRAIL [33]. In relationship with the potential vasoprotective activity of TRAIL [15]–[18], it remains to be established whether the drop of TRAIL after partum associated to stressful conditions represents a risk for future cardiovascular disease, which, anyhow, is rare at the age of the women enrolled in our study. The original aim of the present prospective study was focused on gestation/labor, and no follow-up was planned for the enrolled women. In this respect, at least several years follow-up would be necessary to verify the hypothesis of an increased chance of cardiovascular disease in relation to higher decline of TRAIL levels after delivery.

The last important conclusion of our study is the existence of a strong inverse correlation between CRP and TRAIL. This inverse relationship was observed both at the time of delivery as well as considering the group of women with reduction of TRAIL values ≤20 pg/ml. Moreover, while the inverse association between TRAIL and total cortisol, TRAIL and glycemia and TRAIL and insulin were lost in multivariate analysis considering all the biochemical data, the only variable which showed a persistent association with reduced levels of TRAIL was CRP. In this respect, previous studies concerning the potential association of TRAIL and CRP have been conflicting since TRAIL has been either positively [34], [35] or negatively [36], [37] associated to CRP. Our current data unequivocally show the existence of an inverse association between CRP and TRAIL during pregnancy. In agreement with our data showing an increase of CRP levels during pregnancy with a peak at delivery, previous studies have also shown that CRP increases during pregnancy under both normal and pathological conditions [22]–[25]. Interestingly, it has been proposed that CRP level during pregnancy is a predictor of increased atherogenesis in children [24], a finding particularly interesting in light of the anti-atherosclerotic activity of TRAIL [17], [21], [37], [38].

We are aware that an important limitation of this exploratory analysis is the lack of a critical time point in the third trimester approaching parturition. This does not allow to know how the concentrations of circulating TRAIL change towards the end of pregnancy. Although the available data suggest that reduced circulating TRAIL level (compared to the level in early pregnancy) is associated with stressful conditions, at the moment remains to be defined whether the TRAIL level is already reduced prior to parturition (and thus could be used as an indication of potentially complicated deliveries), or if the TRAIL reduction is caused by stressful conditions during delivery. In spite of these limitations, this study demonstrated for the first time the existence of a significant association between stressful labor conditions and down-regulation of TRAIL after partum.

## Supporting Information

Figure S1 **Correlations between serum levels of TRAIL and biochemical markers measured at delivery.** Inverse correlation between serum levels of TRAIL and cortisol (**A**), between TRAIL and glucose (**B**), between TRAIL and Insulin (**C**) and between TRAIL and CRP (**D**) in women at the moment of delivery (T.3). Correlation coefficient (R) and p values are indicated.
(TIF)

Figure S2 **Serum TRAIL levels with respect to cortisol measured in cord blood.** Serum levels of TRAIL were analyzed in cord blood samples divided based on either low (normal) or high (17 µg/dl) levels of total cortisol. Horizontal bars are median, upper and lower edges of box are 75th and 25th percentiles, lines extending from box are 10th and 90th percentiles.
(TIF)

Table S1 **Bivariate analysis of factors associated with the outcome: TRAIL (T.1–T.3) >20 pg.** SD: standard deviation; *p value calculated with Mann Whitney if independent variable is continuous, and with the two tailed exact Fisher test if independent variable is dichotomous (p value<0.05 in bold). BMI: Body Mass Index; CS: caesarean section; BE: base excess. **Fetal distress if one of the following is true: Apgar at 1^st^ min. >5, Apgar at 5^th^ min. <7, pH<7.1, BE<−12, need of resuscitation in first 30 min., admission to Intensive Neonatal Care.
(DOC)

Table S2 **Biochemical measurements in the study population (n = 73).** Values are given as median (mean±SD). *p<0.01 with respect to T.1, T.2 and cord blood; § p<0.01 with respect to T.1 and cord blood.
(DOC)

Table S3 **Multivariate logistic regression analysis of factors associated with the outcome: TRAIL delivery (T.3)-first quartile.** OR: Odds Ratio; CI: Confidence Interval; *p value<0.05 in bold.
(DOC)
