# Supplementary figures and images for: Circulating TRAIL Shows a Significant Post-Partum Decline Associated to Stressful Conditions
Source: PLoS One. 2011 Dec 14;6(12):e27011. doi: 10.1371/journal.pone.0027011 (PMC3237411; doi:10.1371/journal.pone.0027011)

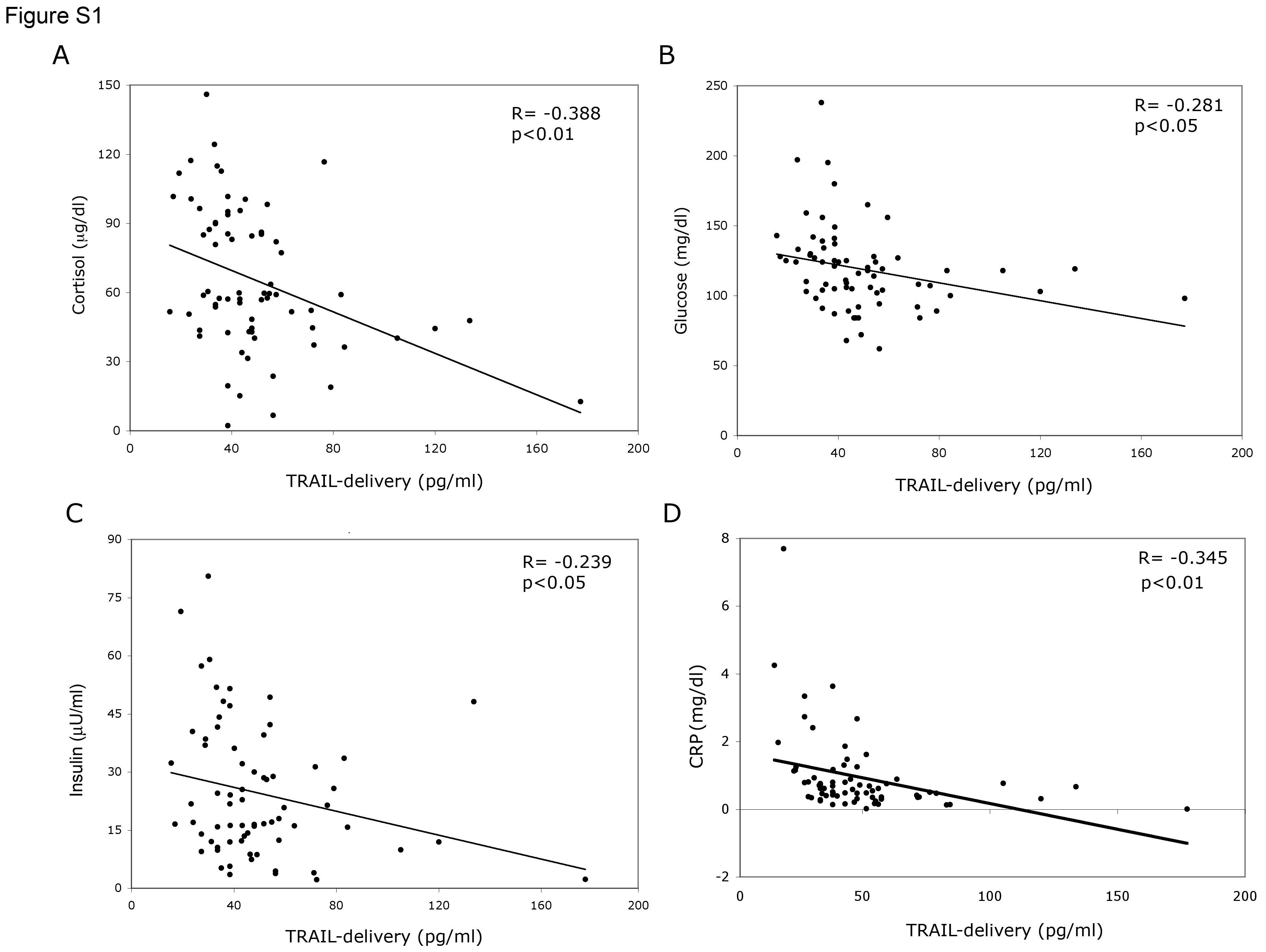

Supplement: Figure S1 — Correlations between serum levels of TRAIL and biochemical markers measured at delivery. Inverse correlation between serum levels of TRAIL and cortisol (A), between TRAIL and glucose (B), between TRAIL and Insulin (C) and between TRAIL and CRP (D) in women at the moment of delivery (T.3). Correlation coefficient (R) and p values are indicated. (TIF) [file pone.0027011.s001.tif]

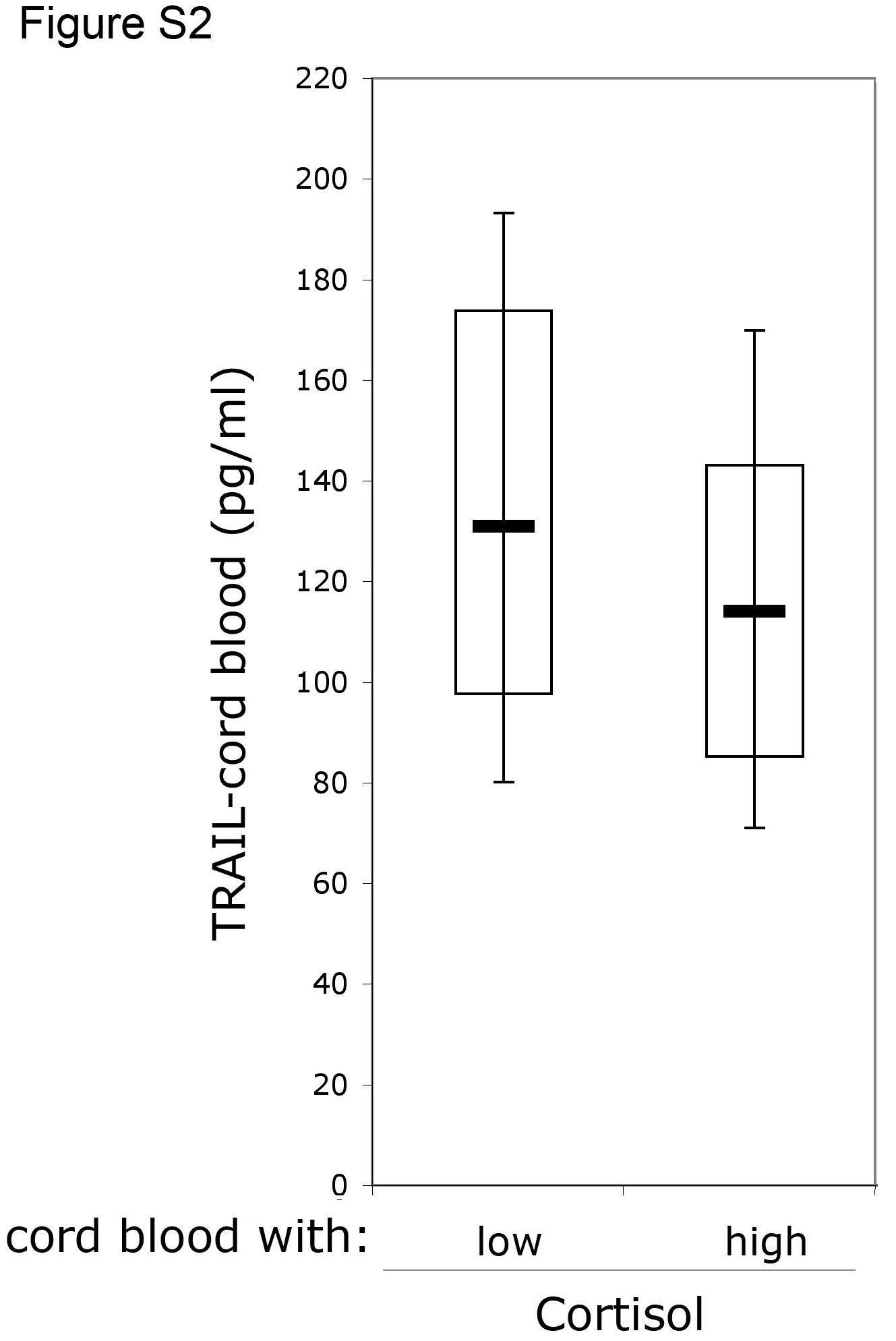

Supplement: Figure S2 — Serum TRAIL levels with respect to cortisol measured in cord blood. Serum levels of TRAIL were analyzed in cord blood samples divided based on either low (normal) or high (17 µg/dl) levels of total cortisol. Horizontal bars are median, upper and lower edges of box are 75th and 25th percentiles, lines extending from box are 10th and 90th percentiles. (TIF) [file pone.0027011.s002.tif]
