# Supplementary material for: Circulating TRAIL Shows a Significant Post-Partum Decline Associated to Stressful Conditions
Source: PLoS One. 2011 Dec 14;6(12):e27011. doi: 10.1371/journal.pone.0027011 (PMC3237411; doi:10.1371/journal.pone.0027011)
Supplement: Table S1 — Bivariate analysis of factors associated with the outcome: TRAIL (T.1–T.3) >20 pg. SD: standard deviation; *p value calculated with Mann Whitney if independent variable is continuous, and with the two tailed exact Fisher test if independent variable is dichotomous (p value<0.05 in bold). BMI: Body Mass Index; CS: caesarean section; BE: base excess. **Fetal distress if one of the following is true: Apgar at 1st min. >5, Apgar at 5th min. <7, pH<7.1, BE<−12, need of resuscitation in first 30 min., admission to Intensive Neonatal Care. (DOC) [file pone.0027011.s003.doc]

**Supplementary** **Table 1. Bivariate analysis of factors associated with the outcome:**

**TRAIL (T.1-T.3) > 20 pg**

|  | **(TRAIL T.1-T.3) ≤20 pg**  **number (%) or**  **mean (SD)** | **(TRAIL T.1-T.3) >20 pg**  **number (%) or**  **mean (SD)** | **p*** |
| --- | --- | --- | --- |
| Maternal age at delivery | 37.3 (2.0 SD) | 38.7 (3.5 SD) | **0.026** |
| Parity  - nulliparous  - pluriparous | 14 (33 %)  28 (67 %) | 21 (70 %)  9 (30 %) | **0.004** |
| Pre pregnancy BMI  BMI categories  - BMI < 30  - BMI ≥ 30 | 22.5 (3.5 SD)  41 (98 %)  1 (2 %) | 23.3 (4.6 SD)  27 (90 %)  3 (10 %) | 0.660  0.301 |
| Conception  - spontaneous  - artificial | 42 (100.0 %)  0 (0.0 %) | 28 (93 %)  2 (7 %) | 0.170 |
| Ethnicity  - Caucasian  - Other | 41 (98 %)  1 (2 %) | 29 (97 %)  1 (3 %) | 1.000 |
| Smoking at 12 weeks gestation  - No  - Yes | 41 (98 %)  1 (2 %) | 29 (97 %)  1 (3 %) | 1.000 |
| Pathological course of pregnancy  - No  - Yes | 36 (86 %)  6 (14 %) | 24 (80 %)  6 (20 %) | 0.539 |
| Gestational Hypertensive disorders  - No  - Yes | 39 (93 %)  3 (7 %) | 28 (93 %)  2 (7 %) | 1.000 |
| Diabetes  - No  - Gestational diabetes | 40 (95 %)  2 (5 %) | 29 (97 %)  1 (3 %) | 1.000 |
| Fetal growth restriction above 10%  - No  - Yes | 22 (54 %)  20 (46 %) | 19 (63 %)  11 (37 %) | 0.470 |
| Gestational age at delivery | 39 (1.4) | 39 (1.5) | 0.704 |
| Induction of labor  - No  - Yes | 38 (93 %)  3 (7 %) | 20 (67 %)  10 (33 %) | **0.011** |
| Delivery modality   - spontaneous vaginal or elective CS - urgent CS or operative vaginal | 39 (93 %)  3 (7 %) | 20 (67 %)  10 (33 %) | **0.011** |
| Analgesia during labor  - Yes  - No | 8 (20 %)  33 (81 %) | 13 (43 %)  17 (57 %) | **0.038** |
| Umbilical pH  - pH ≥ 7.1  - pH < 7.1 | 33 (100 %)  0 (0 %) | 27 (93 %)  2 (7 %) | 0.215 |
| Umbilical BE  - BE ≥ -12  - BE < -12 | 33 (100 %)  0 (0 %) | 26 (96 %)  1 (4 %) | 0.450 |
| Apgar 1^st^ minute  - Apgar ≥ 5  - Apgar < 5 | 42 (100 %)  0 (0 %) | 28 (93 %)  2 (7 %) | 0.170 |
| Apgar 5^th^ minute  - Apgar ≥ 7  - Apgar < 7 | 42 (100 %)  0 (0 %) | 29 (97 %)  1 (3 %) | 0.417 |
| Need of resuscitation in first 30 min.  - No  - Yes | 40 (98 %)  1 (2 %) | 29 (97 %)  1 (3 %) | 1.000 |
| Admission Intensive Neonatal Care  - No  - Yes | 42 (95 %)  2 (5 %) | 24 (80 %)  6 (20 %) | 0.060 |

| Fetal distress**  - No  - Yes | 41 (98 %)  1 (2 %) | 24 (80 %)  6 (20 %) | **0.018** |
| --- | --- | --- | --- |

SD: standard deviation; *p value calculated with Mann Whitney if independent variable is continuous, and with the two tailed exact Fisher test if independent variable is dichotomous (p value <0.05 in bold). BMI: Body Mass Index; CS: caesarean section; BE: base excess. **Fetal distress if one of the following is true: Apgar at 1^st^ min. >5, Apgar at 5^th^ min. <7, pH <7.1, BE <-12, need of resuscitation in first 30 min., admission to Intensive Neonatal Care.
