# Supplementary material for: Circulating TRAIL Shows a Significant Post-Partum Decline Associated to Stressful Conditions
Source: PLoS One. 2011 Dec 14;6(12):e27011. doi: 10.1371/journal.pone.0027011 (PMC3237411; doi:10.1371/journal.pone.0027011)
Supplement: Table S2 — Biochemical measurements in the study population (n = 73). Values are given as median (mean±SD). *p<0.01 with respect to T.1, T.2 and cord blood; § p<0.01 with respect to T.1 and cord blood. (DOC) [file pone.0027011.s004.doc]

**Supplementary Table 2. Biochemical measurements in the study population (n=73)**

| **Serum**  **samples** | **TRAIL**  **(pg/ml)** | **Cortisol**  **(μg/dl)** | **Glucose**  **(mg/dl)** | **Insulin**  **(μU/ml)** | **CRP**  **(mg/dl)** |
| --- | --- | --- | --- | --- | --- |
| Gestation:  12 weeks (T.1)  16 weeks (T.2) | 63.3 (67.6±27.6)  62.9 (64.0±16.2) | 8 (9.4±8.6)  22.2 (24±9.6) | 95 (96.9±14.3)  90 (92.7±16.3) | 9.3 (16.3±17.3)  11.7 (23.4±27.8) | 0.24 (0.40±0.45)  0.31 (0.41±0.33) |
| Delivery (T.3) | 43.3 (49.3±26.4)* | 58.8 (65.1±30.4)* | 116 (118.6±29.7)* | 20.8 (24.7±16.9)§ | 0.58 (0.92±1.16)* |
| Cord blood | 125.7 (131.6±52.0) | 13.5 (16.5±9.81) | 90.0 (90±25.2) | 6.8 (7.12±6.24) | 0.01 (0.06±0.25) |

Values are given as median (mean±SD).

*p<0.01 with respect to T.1, T.2 and cord blood; § p<0.01 with respect to T.1 and cord blood.
