# Supplementary material for: Circulating TRAIL Shows a Significant Post-Partum Decline Associated to Stressful Conditions
Source: PLoS One. 2011 Dec 14;6(12):e27011. doi: 10.1371/journal.pone.0027011 (PMC3237411; doi:10.1371/journal.pone.0027011)
Supplement: Table S3 — Multivariate logistic regression analysis of factors associated with the outcome: TRAIL delivery (T.3)-first quartile. OR: Odds Ratio; CI: Confidence Interval; *p value<0.05 in bold. (DOC) [file pone.0027011.s005.doc]

**Supplementary Table 3. Multivariate logistic regression analysis of factors associated with the outcome: TRAIL delivery (T.3)-first quartile**

|  | **OR** | **95% CI** | **p*** |
| --- | --- | --- | --- |
| Cortisol T.3 | 1.025 | 0.987-1.063 | 0.198 |
| Insulin T.3 | 1.027 | 0.974-1.082 | 0.321 |
| Glycemia T.3 | 1.005 | 0.974-1.035 | 0.765 |
| CRP T.3 | 4.034 | 1.604-10.147 | **0.003** |

OR: Odds Ratio; CI: Confidence Interval; *p value <0.05 in bold.
